# Germline DNA Damage Repair Gene Alterations in Patients with Metachronous Breast and Colorectal Cancer

**DOI:** 10.3390/ijms251910275

**Published:** 2024-09-24

**Authors:** Rolando André Rios Villacis, Luiza Côrtes, Tatiane Ramos Basso, Luisa Matos do Canto, Jeferson Santos Souza, Mads Malik Aagaard, Maria Nirvana da Cruz Formiga, Samuel Aguiar, Maria Isabel Achatz, Silvia Regina Rogatto

**Affiliations:** 1 Department of Clinical Genetics, University Hospital of Southern Denmark, Beriderbakken 4, 7100 Vejle, Denmark; rolando.andre@unb.br (R.A.R.V.); luiza.cortes@unesp.br (L.C.); tatianebasso2015@gmail.com (T.R.B.); luisa.matos.do.canto.alvim@rsyd.dk (L.M.d.C.); mads.jorgensen@rsyd.dk (M.M.A.); 2 Department of Genetics and Morphology, Institute of Biological Sciences, University of Brasília-UnB, Brasília 70910-900, DF, Brazil; 3 Tocogynecology Graduation Program, Medical School, São Paulo State University UNESP, Botucatu 18618-687, SP, Brazil; 4 Health Technology Institute, SENAI CIMATEC, Salvador 41650-010, BA, Brazil; jeferson.s.souza@unesp.br; 5 Department of Oncogenetics and Clinical Oncology, A.C. Camargo Cancer Center, São Paulo 01509-010, SP, Brazil; nirvanaformiga@gmail.com; 6 Colorectal Cancer Reference Center, A.C. Camargo Cancer Center, São Paulo 01509-010, SP, Brazil; samuel.aguiar.jr@gmail.com; 7 Cancer Genetics Unit, Oncology Branch, Hospital Sirio-Libanês, São Paulo 01308-050, SP, Brazil; miachatz@mochsl.org.br; 8 Institute of Regional Health Research, Faculty of Health Sciences, University of Southern Denmark, 5000 Odense, Denmark; 9 Danish Colorectal Cancer Center South, 7100 Vejle, Denmark; 10 Botucatu Medical School Hospital, São Paulo State University UNESP, Botucatu 18618-687, SP, Brazil

**Keywords:** hereditary cancer, germline variants, colorectal cancer, breast cancer, whole exome sequencing, cancer predisposition

## Abstract

A hereditary component of breast (BC) and colorectal cancer (CRC) has been described in approximately one-third of these tumor types. BC patients have an increased risk of developing CRC as a second primary tumor and vice versa. Germline genomic variants (NextSeq550, Illumina) were investigated in 24 unrelated BC and/or CRC patients and 7 relatives from 3 index patients. Fifty-six pathogenic or likely pathogenic variants were identified in 19 of 24 patients. We detected single-nucleotide variants (SNVs) in CRC predisposition genes (*MLH1* and *MUTYH*) and other promising candidates (*CDK5RAP3*, *MAD1L1*, *NOS3*, and *POLM*). Eighteen patients presented SNVs or copy number variants (CNVs) in DNA damage repair genes. We also identified SNVs recently associated with BC or CRC predisposition (*PABPC1*, *TYRO3*, *MAP3K1*, *SLC15A4*, and *LAMA1*). The *PABPC1*c.1255C>T variant was detected in nine unrelated patients. Each patient presented at least one SNV/CNV in a candidate gene, and most had alterations in more than one gene, reinforcing a polygenic model for BC/CRC predisposition. A significant fraction of BC/CRC patients with a family history of these tumors harbored deleterious germline variants in DNA repair genes. Our findings can lead to strategies to improve the diagnosis, genetic counseling, and treatment of patients and their relatives.

## 1. Introduction

Breast and colorectal cancer (BC and CRC) are among the three most frequent cancers worldwide, with an incidence of at least 10% each in 2020 [1]. Of interest is that, in the last decade, the diagnosis of CRC in young patients (less than 50 years of age) has increased significantly [2].

Patients with BC have a higher risk of developing CRC as a second primary malignancy [3], and the opposite is also true [4]. The co-occurrence of BC and CRC has been reported in some families [5,6,7,8]. One of the main risk factors associated with the development of multiple primary tumors is mutation in hereditary cancer predisposition genes [3,9]. Although 20–30% of BC and CRC patients seem to have a familial or heritable cancer-associated component, only 5–10% are classified as hereditary due to the presence of pathogenic variants in well-known high-penetrance genes [10,11]. 

Hereditary BC is mainly caused by germline variants in the *BRCA1*/*2* and *TP53* genes, which are the most prevalent genes related to Hereditary Breast and Ovarian Cancer (HBOC) and Li–Fraumeni syndrome (LFS), respectively [12,13]. In addition to *BRCA1/2*, multigene panels have revealed that *PTEN*, *STK11*, *CDH1*, *PALB2*, *BRIP1*, *ATM*, *BARD1*, *CHEK2*, *NBN*, *NF1*, *RAD51C*, and *RAD51D*, among others, are associated with breast and ovarian cancer risk [14,15]. Patients with HBOC are at increased risk not only of BC but also of other cancer types, including CRC [16,17]. On the other hand, hereditary CRC patients related to Lynch syndrome (LS) have predominantly germline variants in DNA mismatch repair (MMR) genes (*MLH1*, *MSH2*, *MSH6*, *PMS2*, and *EPCAM* deletion) [18,19]. The risk of BC in the LS context remains controversial. A systematic review revealed that 8 of 21 studies demonstrated statistical evidence of an association between BC risk (2- to 18-fold compared to the general population) and LS [20]. Sheehan et al. [21] suggested that LS patients with *PMS2* pathogenic germline variants have a higher risk of BC development. Recently, Schwartz et al. [22] evaluated 272 LS patients, of which 13 presented primary BC and *MLH1*, *MSH2*, *MSH6*, or *PMS2* pathogenic/likely pathogenic variants. According to the authors, the occurrence of BC in LS patients is probably a sporadic event. However, the authors described that a small set of LS patients who developed BC had *MLH1*, *MSH2*, *MSH6*, or *PMS2* germline variants, suggesting that mismatch repair genes should be tested in these patients.

In the last decade, the extensive use of next-generation sequencing (NGS), mainly whole exome sequencing (WES), allowed for the discovery of germline variants in genes potentially associated with the risk of developing BC or CRC, such as *CHEK2*, *PALB2*, *MSH6*, *ROBO1*, biallelic *MUTYH*, *FAN1*, *MAP3K1*, and *SLC15A4* [11,23,24,25,26,27]. Additionally, copy number variations (CNVs) may help explain the missing heritability of hereditary cancers with no pathogenic variants in well-known predisposition genes [7]. Previously, our group used microarray technology and identified a germline deep intronic deletion of *ROBO1* in three patients with hereditary BC and CRC [7]. Further analysis revealed that this deletion affected gene expression, and in two families, co-segregation of the variants was detected, thus supporting the role of *ROBO1* in BC/CRC risk [7]. More recently, we described germline CNVs (one duplication and two deletions) from WES analysis encompassing *PMS2* in three unrelated patients with BC and CRC [8]. 

Despite the advances in NGS technology and interpretation of its results, the genetic component of many cases of hereditary BC and/or CRC remains unexplained, compromising genetic counseling, management, and treatment of the patients [10,19]. A few studies have explored the germline profile of patients with metachronous BC/CRC and their association with family cancer history [7,8,25]. Herein, we investigate germline variants and CNVs in patients diagnosed with breast and/or colorectal cancer, most of them with a positive family history of these two tumor types, who tested negative for the most common hereditary cancer genes related to the phenotype. We aimed to identify germline variants associated with BC/CRC risk.

## 2. Results

### 2.1. Clinical and Pathological Data of the Patients

**Figure 1:**
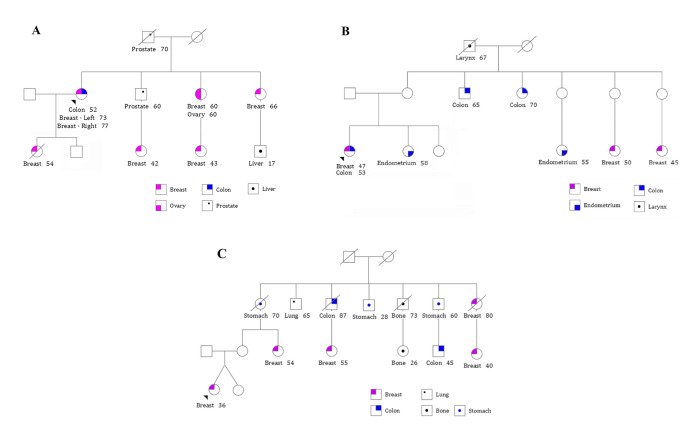
Pedigree of families from index patients MPT19, MPT21, and MPT23 investigated by whole exome sequencing (WES): (**a**) three relatives of MPT19: daughter with BC (III-1), brother with prostate cancer (II-3), and son (III-2) with no cancer; (**b**) two sisters of MPT21, one with endometrial cancer (III-2) and another unaffected by cancer (III-3); (**c**) two relatives of MPT23 with no cancer: twin sister (IV-2) and mother (III-2).

Fifteen index cases presented both BC and CRC, seven had BC, and two had CRC. Eleven index patients reported a family cancer history of CRC and BC, while only one patient (MPT1) had no family history of cancer. Twenty-three patients fulfilled at least one referral clinical criteria for hereditary cancer syndromes: thirteen patients presented HBOC [28], one LS (MPT12) [29], and eight fulfilled the criteria for both cancer syndromes. Moreover, 20 patients fulfilled the clinical criteria for Hereditary Breast and Colorectal Cancer (HBCC) [30,31], and two (MPT8 and MPT23) met the clinical criteria for LFS (Chompret criteria) [32]. Supplementary Table S1 summarizes the patients’ clinical and pathological data, family history of cancer, and the treatment received. Family members of three index cases (MPT19, MPT21, and MPT23) agreed to participate in this study and were evaluated by WES (Figure 1). Three relatives of MPT19 were included: two with cancer (MPT19.2: daughter with BC, MPT19.5: brother with prostate cancer) and one son (MPT19.4) with no cancer (Family 1). Patient MPT21 had two sisters tested by WES, one with endometrial cancer (MPT21.2) and the other unaffected by cancer (MPT21.3) (Family 2). Two relatives (MPT23.2: twin sister and MPT23.3: mother) of index case MPT23, who are currently unaffected by cancer, were also sequenced (Family 3).

### 2.2. Single-Nucleotide Variants (SNVs) and InDels

A total of 56 pathogenic (P) or likely pathogenic (LP) SNVs were identified in our cohort (Supplementary Table S2). Patients MPT19 and MPT21 presented the largest number of SNVs, with 8 and 12, respectively, while five patients (MPT1, MPT2, MPT5, MPT9, and MPT12) had no P/LP germline variants. Interestingly, we identified two LP variants in genes previously associated with CRC risk: *MUTYH* (MPT6) and *MLH1* (MPT11). Three LP variants were detected in more than one patient: *ARSB* c.944G>A, *GNRHR* c.317A>G (MPT15 and MPT16), and *SPG7* c.1529C>T (MPT6 and MPT19). 

Members of the three families investigated by WES shared several P/LP variants with the index cases (Supplementary Table S3). Variants in *KIRREL1*, *TMEM144*, and *ZDHHC11B* were shared between MPT19 and her three relatives (Family 1). *RP1* c.2212-1G>A was the only variant co-segregating with the disease. Variants in four genes (*ROPN1*, *PRSS3*, *BPIFB1*, and *BCORL1*) were detected in all individuals from Family 2, while four (*TUBB2B*, *CAFAP54*, *PSG2*, and *SIRPB1*) were exclusive of the MPT21 index case and her relative with cancer (MPT21.2). Index patient MPT23 and her relatives (Family 3) presented a pathogenic variant in *NOS3*. Except for *MLH1*, no other P/LP gene variants, including those identified in the relatives, were considered clinically actionable [33,34,35].

We identified 64 variants of uncertain significance (VUSs) with a potential role in the phenotype in all patients except MPT10 (Supplementary Table S4). At least three unrelated index patients presented variants in *PABPC1*, *TTN*, *TDG*, *NOC3L*, and *CRY2*. Nine index patients presented *PABPC1* c.1255 C>T. Interestingly, this variant was found in the COSMIC database. VUSs in seven other genes (*ATM*, *CSMD3*, *KDR*, *ERCC3*, *PDE4DIP*, *EPHA3*, and *MYC*) were also described in COSMIC and/or CIViC databases (Supplementary Table S4). Two patients exhibited variants in well-known cancer risk genes, *ATM* c.5558A>T (MPT2) and *MLH1* c.1853A>T (MPT11). Three unrelated index patients (MPT1, MPT3, and MPT5) presented the *TDG* c.602A>C variant. Three patients presented potentially damaging VUSs in other DNA repair genes, such as *ERCC3* (MPT12), *XPA* (MPT23), and *XPC* (MPT19). Overall, 13 index cases presented SNVs (P/LP or VUS) in DNA repair genes (Table 1).

**Figure 2:**
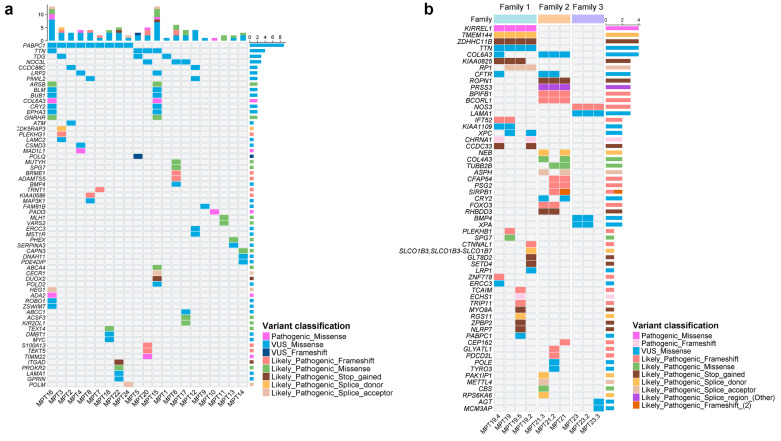
Mutational landscape of germline single-nucleotide variants (pathogenic, likely pathogenic, and prioritized VUS). Oncoprint charts display single-nucleotide variants in (**a**) 21 index patients and (**b**) relatives of three index patients (MPT19, MPT21, and MPT23). The genes are ordered from top to bottom by decreasing the number of altered samples (right panel). The top panel describes the number of altered genes per sample, which is indicated at the bottom of each image. Color-coded annotations highlight the presence and classification of each variant.

A total of 23 VUSs were identified in relatives of the three families investigated (Supplementary Table S5). All relatives of patient MPT19 showed *TTN* c.96235G>A. All individuals from Families 2 and 3 shared variants in *COL6A3* and *LAMA1*, respectively. Figure 2a graphically displays the genes affected by P, LP, and selected VUSs in all index cases except those with relatives tested. Figure 2b is representative of the SNVs found in the index patients and their relatives evaluated by WES.

### 2.3. Copy Number Variations (CNVs)

We prioritized 87 CNVs among the 24 cases evaluated by WES (Supplementary Table S6). Although all except one patient (MPT3) presented CNVs, four index cases (MPT7, MPT17, MPT21, and MPT22) did not fulfill the adopted criteria to consider the CNVs potentially damaging. Most of the CNVs prioritized were classified as VUSs. We also found five pathogenic or likely pathogenic CNVs: loss of *RECQL4* in MPT6; gain of the short arm of chromosome X (26157103-30578472), covering 14 genes, in MPT8; loss of both *NRXN1* and *TTN* in MPT3; and a gain of ~62 Mb on chromosome 4 of patient MPT24 (Supplementary Table S6). The most common genes affected by CNVs, in at least three different cases, were *CENPE* (two gains and one loss), *MUC19* (four duplications and two deletions), *TTN* (two gains and five losses), and *TYRO3* (five duplications). Notably, ten patients showed CNVs covering DNA repair genes (Table 1). Five HBCC patients (MPT2, MPT6, MPT11, MPT12, and MPT23) presented both SNVs and CNVs affecting DNA repair genes.

**Figure 3:**
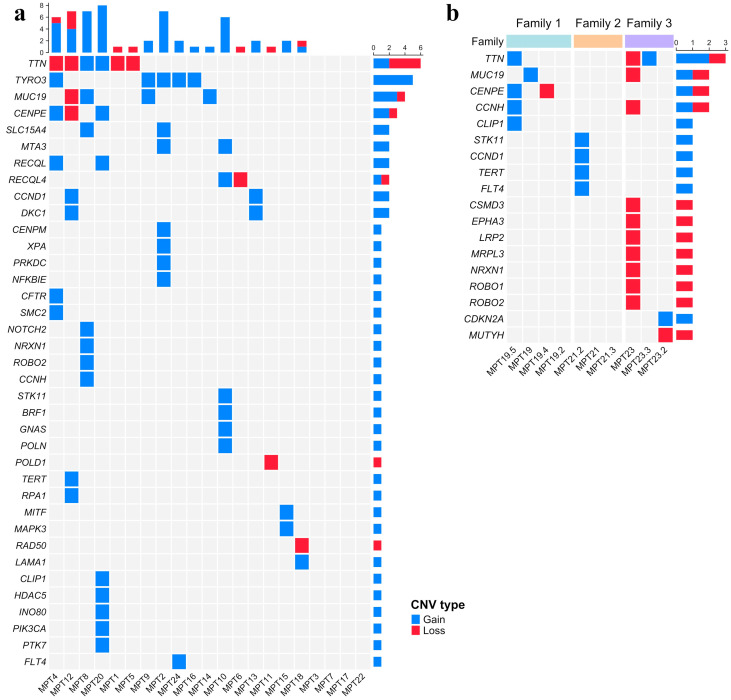
Oncoprint charts showing the prioritized copy number variations identified in (**a**) 21 index patients and (**b**) relatives of index patients (MPT19, MPT21, and MPT23) tested by whole exome sequencing (**b**). The genes are ordered from top to bottom by decreasing the number of altered samples (right panel), while the top panel describes the number of altered genes per sample. Individualized patients are shown at the bottom of each column. Duplications and deletions are represented by blue and red, respectively.

More than one individual per family presented *CENPE* and *TTN* genes covered by CNVs. A loss of *TTN* was detected in MPT23, while her mother presented a duplication encompassing the same gene. A loss and a gain of *CENPE* were identified in two relatives of patient MPT19, MPT19.4 and MPT19.5, respectively (Supplementary Table S7). The prioritized genes affected by CNVs are shown in Figure 3a. Figure 3b also exhibits the genes affected by CNVs in the tested relatives. Thirteen cases (~54%) presented prioritized CNVs mapped in common fragile sites (Supplementary Table S8), with seven affecting the fragile site, FRA2G, which encompasses the *TTN* gene. 

## 3. Discussion 

Epidemiological data have demonstrated an increased risk of BC patients developing CRC as a second primary tumor and vice versa [3,4]. Nevertheless, this phenotype remains poorly explored [7,8,25]. Herein, we evaluated germline variants (SNVs and CNVs) in 24 patients with BC and/or CRC; all except one index case fulfilled the criteria for hereditary cancer predisposition syndromes. We also evaluated relatives of three index cases that agreed to participate in this study.

The WES analysis revealed P and LP SNVs in 19 of 24 index patients (~79%). However, only a subset of these variants is potentially associated with an increased risk of cancer development. Patient MPT11 presented two variants (LP and VUS) in *MLH1* associated with LS, while in MPT6, the monoallelic *MUTYH* c.1187G>A (LP) was found. *MUTYH* codifies a base excision repair (BER) protein, and one P/LP variant without a polyposis phenotype showed a moderate risk for CRC development [19].

We also detected CNVs with a potential impact on BC/CRC risk. Recently, we reported that three HBOC patients with BC and CRC presented CNVs in *PMS2* and *POLE2* [8]. Herein, we identified CNVs covering two well-known genes associated with CRC predisposition: *POLD1* (MPT11) and *STK11* (MPT10) [19]. A pathogenic gain mapped in 4q28-q35 (191 genes) was detected in MPT24, who had no phenotype of a known predisposition to cancer syndrome. However, several candidate genes mapped in this region might contribute to cancer risk.

Notably, eighteen patients (75%) harbored SNVs or CNVs in DNA damage repair genes belonging to different pathways (BER: base excision repair, NER: nucleotide excision repair, MMR: mismatch repair, HR: homologous recombination repair, NHEJ: non-homologous end-joining repair). Eleven cases showed at least two germline alterations in genes of these pathways, while five patients (MPT4, MPT8, MPT10, MPT18, and MPT20) presented only CNVs in DNA repair genes. Three index cases (MPT1, MPT3, and MPT5) were carriers of *TDG* c.602A>C (VUS), a gene that belongs to the BER pathway. Germline *TDG* variants were associated with CRC susceptibility [36,37].

Nucleotide excision repair (NER) is a mechanism to remove helix-distorting DNA lesions by repairing bulky DNA lesions. We identified eight index patients with SNVs or CNVs in NER genes: *ERCC3* (MPT12), *XPC* (MPT19), *XPA* (MPT23, MPT2), *CCNH* (MPT8, MPT23), and *CRY2* (MPT15, MPT16, MPT21). Rare germline variants in *ERCC3*, *XPA*, and *XPC* are related to xeroderma pigmentosum, a disease that increases the appearance of ultraviolet-induced skin cancer at an early age. *ERCC3* was described as a candidate gene for HBOC predisposition [38,39]. Common variants of *XPA* and *XPC* genes have been linked to BC and CRC susceptibility [40,41]. Previously, the *CCNH* damaging variant was identified in a *BRCA1*/2-negative BC patient [42]. *CRY2* is an essential circadian clock component that regulates the transcription of DNA repair genes, mainly those from the NER pathway [43]. Missense variants of *CRY2* can suppress TP53 and promote cell growth [43].

Eleven individuals presented SNVs/CNVs in genes associated with double-strand break repair, such as *ATM* c.5558A>T in case MPT2. ATM is essential for initiating double-strand break repair by HR. *ATM* mutations have a moderate risk for CRC and a two-fold increased risk for ductal BC (P variants) [44]. Previously, we described *ATM* variants in 17% (12 cases) of early-onset CRC patients [45]. Two patients (MPT15 and MPT16) presented *BLM* c.543C>A. This gene codifies the RecQ DNA helicase involved in the HR pathway. Deleterious germline *BLM* variants were found in BC and CRC families [46,47]. Patient MPT18 had a gain of *RAD50*, which participates in the HR pathway and is considered a candidate for BC risk [48]. *ZSWIM7* (c.336T>G; MPT16) is involved in HR regulation and is a new candidate gene for hereditary polyposis [49]. Two patients (MPT6 and MPT10) showed duplications encompassing *RECQL4*, a candidate gene for hereditary BC [42]. Moreover, index patients MPT4 (*RECQL*) and MPT20 (*RECQL* and *INO80*) presented CNVs in candidate genes related to CRC risk [47,50].

Six patients presented SNVs/CNVs in DNA polymerase genes: *POLD1* (MPT11), *POLD2* (MPT15), *POLQ* (MPT5), *POLM* (MPT24), *POLN* (MPT10), and *PRKDC* (MPT2). Although *POLD2* has not been directly associated with CRC risk, it codifies a subunit of the holoenzyme DNA polymerase delta, which is essential for DNA replication and repair. *POLD1*, a well-known gene associated with CRC predisposition, codifies the catalytic subunit of the same polymerase [19]. Therefore, *POLD2* is a potential cancer predisposition gene. Potentially pathogenic variants in *POLQ* (DNA polymerase theta) and *POLN* (polymerase nu) were reported in HBOC and hereditary CRC cases [23,51,52]. The *PRKDC* gene (NHEJ pathway) was related to CRC predisposition [53]. The POLM protein is involved in DNA double-strand breaks by NHEJ and is prone to misincorporation, resulting in genomic instability [54]. Although variants in *POLM* have not been directly associated with cancer risk, it is a putative predisposition gene since variants in other DNA polymerase genes (*POLD1* and *POLE*) have already been related to CRC risk [19].

In addition to its crucial role in carcinogenesis, deficiency in DNA repair pathways may indicate specific therapeutic interventions. Patients with HR defects, mainly due to germline variants in *BRCA1/2*, can benefit from using platinum agents and poly ADP-ribose polymerase 1 (PARP1) inhibitors [55]. Interestingly, *BRCA1/2*-positive patients may also benefit from a less-invasive surgical intervention (breast-conserving therapy), which has achieved similar survival rates to mastectomy [56]. Deficiency in MMR genes is often associated with an immune-rich microenvironment, with high CD8 and T-lymphocyte infiltration, which promotes an effective antitumor response and explains the benefits of immune checkpoint blockade regardless of tumor location, particularly anti-PD-1/PD-L1 inhibitors [57,58,59].

In addition to damaging variants found in DNA damage repair genes, we detected P/LP variants in two genes (*CDK5RAP3* and *MAD1L1*) related to cell cycle regulation. Patient MPT3 (BC/CRC) carried *CDK5RAP3* c.988+1G>A (LP). This tumor suppressor gene is implicated in apoptosis, cell proliferation, cell growth, and metastasis. A physical interaction between the CDK5RAP3 protein and BRCA2 was recently reported, suggesting that *CDK5RAP3* is involved in double-strand break repair [60]. Index case MPT4 carried *MAD1L1* c.175C>T (LP). The functional defects of this gene may lead to aneuploidy and chromosomal instability [61].

Three genes (*PABPC1*, *NOC3L*, and *TTN*) presenting potentially damaging variants were detected in at least three patients. *PABPC1*c.1255C>T was identified in nine unrelated patients. This gene codifies a protein involved in different functions related to mRNA metabolism (translation, stability, nonsense-mediated decay, and regulation of miRNA activity). Seven of nine variants of *PABPC1* described in LS patients were located on exon 6 (c.739-1G>A) and may affect one of the four protein domains (RRM3 domain) associated with RNA recognition [62]. Our patients presented the *PABPC1* variant located on exon 9, which codifies part of the protein linker domain with a role in its translation activity [63]. This variant was previously described in the COSMIC database, suggesting its potential role in carcinogenesis. In this context, *PABPC1*c.1255C>T, identified in our patients, is a promising candidate for cancer predisposition. Three patients (MPT6, MPT17, and MPT20) presented *NOC3L* variants, whose gene function is predicted to be involved in DNA replication initiation and adipocyte differentiation. Common variants of *NOC3L* were related to increased risk of CRC and gastric cancer development [64].

Alterations involving the *TTN* gene were frequently detected in our cases (SNV: four cases; CNVs: five cases; SNV+CNV: two cases). Germline variants in *TTN* were previously associated with BC [65,66]. Recently, *TTN* was suggested as a potential therapeutic target for CRC based on gene and protein expression levels [67]. Interestingly, the *TTN* locus is mapped in the fragile site, FRA2G [68]. Eight patients presented genes covered by CNVs mapped in common fragile sites [*CLIP1* (MPT20), *FLT4* (MPT24), *RAD50* (MPT18), *STK11* (MPT10), *RECQL4* (MPT6 and MPT10), and *SLC15A4* (MPT2 and MPT8)]. *CLIP1* and *FLT4* are predisposing candidate genes for HBOC [10,42], while *SLC15A4* was reported to be a new candidate gene for hereditary CRC [24]. CNVs at fragile sites are not caused primarily by DNA repair defects, thus indicating interesting hot spots to search for cancer-related genes [69]. 

Two losses and four gains of *MUC19* were identified in six unrelated patients. A study with seven early-onset CRC cases pinpointed *MUC19*, among others, as a candidate gene for CRC predisposition [70]. The receptor tyrosine kinase 3 (*TYRO3*) was involved in gains in five patients in our cohort. Four of forty-eight patients with familial CRC presented two different SNVs in *TYRO3* [62]. We also identified CNVs covering genes that codify centromere-associated proteins in four patients: *CENPE* (MPT4, MPT12, and MPT20) and *CENPM* (MPT2). These genes were described as potential candidates for CRC predisposition [70,71]. A partial gain of *ROBO1* (MPT8) and losses of *ROBO1* and *ROBO2* (MPT23) were detected in our patients. We previously identified an intronic deletion of *ROBO1* in three patients [7]. Further analyses revealed a reduction in gene expression, and a co-segregation with the disease, thus suggesting this gene as a candidate for BC/CRC predisposition [7].

We also detected potentially damaging variants in a set of genes recently related to BC and/or CRC predisposition in WES studies. Variants in laminin genes were found in three patients: MPT3 (*LMC2*c.1637C>A), MPT22, and MPT23 (*LAMA1*c.2657C>T). *LMC2* and *LAMA1* genes were suggested as novel candidates for HBOC predisposition in Tunisian families [72]. Patient MPT8 harbored *MAP3K1* c.2062C>G. Recently, *MAP3K1*c.175C>T (VUS) was associated with an increased risk of CRC in an Iranian family [11]. We also identified other promising germline gene variants reported in BC patients and families evaluated by WES: *FAM81B* (MPT9), *SERPINA3* (MPT13), and *DNAH11* (MPT14) [42,73,74].

Co-segregation analyses were performed in three families. Index patient MPT19 and four tested relatives presented *KIRREL1*c.1718C>T, *TMEM144* c.802+1G>A, and *ZDHHC11B* c.780C>G. Among them, only *ZDHHC11B* was previously associated with pancreatic cancer risk [75]. In the second family in which MPT21 and two relatives were tested, we found four LP variants (*BPIF1*, *BCORL1*, *ROPN1*, and *PRSS3*) and four other cancer-related genes (*TUBB2B*, *CFAP54*, *PSG2*, and *SIRPB1)*. However, these eight genes were not previously associated with cancer risk or presented biological functions that explain the phenotype. The third family (MPT23 and two cancer-free relatives) presented the LP variant in *NOS3*, which encodes the enzyme endothelial nitric oxide synthase. Single-nucleotide polymorphisms of *NOS3* have been related to BC and CRC risk [76,77]. *TTN* and *LAMA1*, as previously discussed, were identified in all tested relatives of MPT19 and MPT23 patients, respectively. Similarly to SNVs, we investigated the role of CNVs in these three families. *STK11* and *FLT4* genes were covered by CNVs in the relative of MPT21.2, who presented endometrial cancer. Endometrial cancer was described in individuals with *STK11* alterations [78]. A relative of MPT23, MPT23.2 (currently cancer-free), showed pathogenic CNVs of *MUTYH*, therefore requiring clinical monitoring for CRC. Overall, we described a significant number of patients with CNVs affecting genes related to hereditary cancer syndromes.

Although we exhaustively investigated SNVs and CNVs in our patients and the literature data, our study has limitations, including the small number of patients and their relatives (seven agreed to participate). To confirm and validate our findings, it is necessary to conduct studies on a larger cohort of breast and colorectal cancer patients and their family members. The co-segregation analysis is relevant for establishing an association between the presence of variants and the risk of cancer and increases the potential to classify cancer-predisposing genes for which the clinical significance is unknown. Another limitation of our study is the lack of functional assays to support the role of potentially damaging variants. DNA variants can have various effects on protein function, and determining their pathogenicity is a challenging task. The functional assays are difficult to interpret clinically, and only a subset of unclassified variants located in specific domains of the genes could be explored [79]. However, we identified several promising candidate genes for hereditary BC and CRC that should be validated in other cohorts.

## 4. Materials and Methods

### 4.1. Patients

A cohort of 445 cancer patients fulfilling criteria for hereditary cancer syndromes or with familial aggregation of cancer were treated at A.C. Camargo Cancer Center, São Paulo, Brazil (2009–2016). Of those, we selected 24 unrelated patients and 7 relatives from 3 families to investigate for germline variants and CNVs using whole exome sequencing (WES). All patients were negative for *BRCA1*, *BRCA2*, and *TP53* pathogenic variants, which were evaluated by Sanger sequencing. The medical and pathological records were revised to confirm the diagnosis of the primary tumors, therefore ruling out that any of them would be metastasis. All patients were followed at the Oncogenetics Department of the A.C. Camargo Cancer Center. This study was conducted following the ethical guidelines and regulations from the Declaration of Helsinki. Written informed consent was obtained from all patients and family members before blood sample collection. The Brazilian National Research Ethics Commission (CONEP) approved the study (Protocol # 2136/15).

### 4.2. DNA Isolation and Whole Exome Sequencing (WES)

The standard protocol of the QIAamp DNA Blood Mini QIAcube Kit (Qiagen, Hilden, Germany) was used to extract genomic DNA from peripheral blood lymphocytes. The quality/integrity of the nucleic acids was assessed with the Genomic DNA ScreenTape assay (Agilent TapeStation, Agilent Technologies Inc., Santa Clara, CA, USA). DNA quantity was obtained using a Qubit dsDNA BR assay Kit (Thermo Fisher Scientific Inc., Waltham, MA, USA). Before sequencing, libraries were constructed using a TruSeq Rapid Exome Library Prep Kit (Illumina, San Diego, CA, USA), following the manufacturer’s recommendations. The pooled enriched libraries were PCR-amplified and evaluated using the High Sensitivity D1000 ScreenTape assay (Agilent). The exome sequencing was performed as paired-end (2 × 150 bp) on a NextSeq550 System (Illumina).

### 4.3. Data Analysis and SNV and CNV Prioritization

A Genome Analysis Toolkit (GATK) was used to align the sequence reads (Burrows-Wheeler Alignment software package and BWA-MEM algorithm) to the human genome (UCSC hg19) [80]. The alignment statistics were obtained using the Sequence Alignment/Map (SAMtools), Picard, and Binary Alignment Map (BAMtools). In turn, GATK HaplotypeCaller was applied to perform variant calling and merging. We used VarSeq™ software version 2.2.2 (Golden Helix, Inc., Bozeman, MT, US) to annotate and select germline variants. To select novel and/or rare variants, we filtered all candidates with alternate allele frequencies less than 1% in the Genome Aggregation Database (GnomAD v2.1) [81], Genome (v3), Exome Variants (v2), and Online Archive of Brazilian Mutations (ABRaOM): Brazilian Genomic variants [82]. Variants were selected with a threshold strand Bias Fisher’s < 50, quality by depth >1.5, and a Variant Allele Frequency > 0.2. The variants were filtered out if they resulted in synonymous alterations, potentially presented homopolymer artifacts (homopolymer sequence length > 6), or were predicted as benign/likely benign by the American College of Medical Genetics and Genomics (ACMG) or ClinVar classification [83,84]. Sequence data supporting the remaining variants were evaluated in paired BAMs using the Golden Helix GenomeBrowse visualization tool v.2.2.2. We removed variants in the presence of artifacts resulting from PCR amplification, sequencing, or alignment.

We considered and manually curated only nonsynonymous, protein-damaging, and rare variants. P and LP variants were selected using ACMG guidelines (revised in November 2023). The VUSs that were potentially damaging were initially selected using deleterious effects filtering. We selected variants associated with loss of function or splice altering predictions (Ada scores > 0.6) or predicted as damaging in at least three independent functional prediction algorithms, including Sorting Intolerant From Tolerant (SIFT) [85], Polymorphism Phenotyping v2—Polyphen2 HVAR [86], MutationTaster [87], MutationAssessor [88], Functional Analysis through Hidden Markov Models—FATHMM, and FATHMM-MKL Coding [89], or Combined Annotation-Dependent Depletion (CADD) score > 3. The last stage of VUS prioritization involved a literature search of genes, variants, and the risk related to BC and/or CRC. Next, we selected the variants affecting genes presented in two or more patients or genes that encode proteins associated with DNA replication, DNA repair, and cell cycle control. We also considered VUSs in genes previously associated with hereditary cancer or recently suggested as candidates for BC and/or CRC risk in WES studies. Lastly, we compared the list of prioritized VUSs with the Catalogue Of Somatic Mutations In Cancer (COSMIC) and the Clinical Interpretation of Variants in Cancer (CIViC) to explore the potential role of these alterations in cancer emergence and development [90,91].

WES data were also used to evaluate CNVs through the CNV caller on target regions algorithm (Golden Helix VarSeq v.2.2.2). CNVs with a frequency ≥ 0.01 on the Database of Genomic Variants (DGV) and *p*-value < 0.001 were excluded. The CNV prioritization was performed as described above for genetic variants. Additionally, we investigated if the selected CNVs were mapped in common fragile sites [68,69].

## 5. Conclusions

In summary, we unraveled promising candidate genes for hereditary BC and CRC. Fifty-six P and LP variants were identified in nearly 80% of patients. However, only a set of these variants had the potential to increase the risk of the disease. Notably, we detected 18 patients (75%) with SNVs or CNVs in DNA damage repair genes, which are valuable candidates for BC/CRC predisposition. DNA damage repair genes can modulate cancer risk, progression, and therapeutic response and might be of great relevance in the management of patients with second primary tumors, such as BC/CRC. Genes recently linked to BC and CRC risk were also identified (e.g., *PABPC1*, *MUC19*, *TYRO3*, *MAP3K1*, *SLC15A4*, and *LAMA1*). *PABPC1* c.1255C>T was observed in nine unrelated patients and has a strong potential as a deleterious variant of BC/CRC risk. All cases had at least one alteration in a candidate gene. The presence of more than one candidate gene in several patients reinforces a polygenic model for cancer predisposition in these patients. Although the alterations herein presented need further investigation, we report new candidate genes for cancer risk, which may improve the management and treatment strategies for patients and their relatives.

## Figures and Tables

**Table 1:** DNA repair-related genes altered in our patients.

DNA Repair Pathway	Gene ^a^	Type of Alteration	Case
Base excision repair (BER)	*MUTYH*	SNV (LP)	MPT6
	*TDG*	SNV (VUS)	MPT1, MPT3, and MPT5
Nucleotide excision repair (NER)	*ERCC3*	SNV (VUS)	MPT12
	*XPC*	SNV (VUS)	MPT19
	*XPA*	SNV (VUS)/CNV	MPT2 and MPT23
	*CRY2*	SNV (VUS)	MPT15, MPT16, and MPT21
	*CCNH*	CNV	MPT8 and MPT23
Mismatch repair (MMR)	*MLH1*	SNV (LP/VUS)	MPT11
Homologous recombination (HR)	*ATM*	SNV (VUS)	MPT2
	*BLM*	SNV (VUS)	MPT15 and MPT16
	*ZSWIM7*	SNV (VUS)	MPT16
	*POLQ*	SNV (VUS)	MPT5
	*POLD1* ^a^	CNV	MPT11
	*POLD2* ^a^	SNV (VUS)	MPT15
	*RAD50*	CNV	MPT18
	*RECQL* ^b^	CNV	MPT4 and MPT20
	*RECQL4*	CNV	MPT6 and MPT10
	*POLN*	CNV	MPT10
	*INO80*	CNV	MPT20
	*RPA1* ^c^	CNV	MPT12
Non-homologous end-joining repair (NHEJ)	*POLM*	SNV (LP)	MPT24
	*PRKDC*	CNV	MPT2

^a^ Also participates in NHEJ, BER, NER, MMR, break-induced recombination (BIR), and microhomology-mediated end joining (MMEJ). ^b^ Also participates in NHEJ. ^c^ Also participates in NER.

## Data Availability

The original contributions presented in this study are included in the article/Supplementary Material; further inquiries can be directed to the corresponding author.

## Supplementary Materials

The following supporting information can be downloaded at: https://www.mdpi.com/article/10.3390/ijms251910275/s1.
